# Emergence of Nasal Carriage of ST80 and ST152 PVL+ *Staphylococcus aureus* Isolates from Livestock in Algeria

**DOI:** 10.3390/toxins9100303

**Published:** 2017-09-25

**Authors:** Amir Agabou, Zouleikha Ouchenane, Christelle Ngba Essebe, Salim Khemissi, Mohamed Tedj Eddine Chehboub, Ilyes Bey Chehboub, Albert Sotto, Catherine Dunyach-Remy, Jean-Philippe Lavigne

**Affiliations:** 1 Institut National de la Santé et de la Recherche Médicale, U1047, Université Montpellier, UFR de Médecine, 30908 Nîmes, France; amirveto@gmail.com (A.A.); ngbachristelle@yahoo.fr (C.N.E.); albert.sotto@chu-nimes.fr (A.S.); catherine.remy@chu-nimes.fr (C.D.-R.); 2 Laboratoire PADESCA, Institut des Sciences Vétérinaires, Université des Frères Mentouri-Constantine 1, Constantine 25017, Algérie; tedj.chehboub@gmail.com (M.T.E.C.); ilyesbeycheboub@gmail.com (I.B.C.); 3 Laboratoire de microbiologie, Hôpital Militaire Régional Universitaire de Constantine, Constantine 25001, Algérie; z_ouche@yahoo.fr (Z.O.); salimkhemissi@gmail.com (S.K.); 4 Service des Maladies Infectieuses et Tropicales, CHU Nîmes, 30029 Nîmes, France; 5 Service de Microbiologie, CHU Nîmes, 30029 Nîmes, France

**Keywords:** Algeria, clonal complex, MRSA, MSSA, nasal carriage, Panton–Valentine Leukocidin, *Staphylococcus aureus*, ST80, ST152

## Abstract

The spread of toxinogenic *Staphylococcus aureus* is a public health problem in Africa. The objectives of the study were to investigate the rate of *S. aureus* nasal carriage and molecular characteristics of these strains in livestock and humans in three Algerian provinces. Nasal samples were collected from camels, horses, cattle, sheep and monkeys, as well as humans in contact with them. *S. aureus* isolates were genotyped using DNA microarray. The rate of *S. aureus* nasal carriage varied between species: camels (53%), humans and monkeys (50%), sheep (44.2%), horses (15.2%) and cattle (15%). Nine methicillin-resistant *S. aureus* (MRSA) isolates (7.6%) were identified, isolated from camels and sheep. The *S. aureus* isolates belonged to 15 different clonal complexes. Among them, PVL+ (Panton–Valentine Leukocidin) isolates belonging to ST80-MRSA-IV and ST152-MSSA were identified in camels (*n* = 3, 13%) and sheep (*n* = 4, 21.1%). A high prevalence of toxinogenic animal strains was noted containing TSST-1- (22.2%), EDINB- (29.6%) and EtD- (11.1%) encoding genes. This study showed the dispersal of the highly human pathogenic clones ST152-MSSA and ST-80-MRSA in animals. It suggests the ability of some clones to cross the species barrier and jump between humans and several animal species.

## 1. Introduction

*Staphylococcus aureus* is both an animal- and human-opportunistic bacterium capable of causing a wide range of severe diseases [1]. In humans, *S. aureus* is a common inhabitant of the skin, also colonizing the nares and other mucosa [2]. In animals, the nostrils, nares, mouth and perineum are the principal carriage sites [3]. The transmission of *S. aureus* is by direct contact with contaminated environments (objects and products) and colonized/infected individuals (animals and people), with hands as the main vectors [4]. Infections due to *S. aureus* constitute an important public health problem, notably due to the rise of multidrug resistance with the spread of the methicillin-resistant strains (MRSA) in humans, farm animals and in the wider environment [5]. The alarming zoonotic potential of some *S. aureus* clones is now well recognized, and contact with animals seems to be one of the foremost factors influencing MRSA colonization and infection in human populations [5]. Many investigations have described individuals with regular contact with animals out of household settings to be at high risk of becoming colonized/infected by these strains [6].

In Algeria, several studies have examined the prevalence of *S. aureus* and especially MRSA in patients in healthcare settings. ST80-MRSA IV PVL+ (Panton–Valentine Leukocidin) is the leading clone in this country [7]. Very few data are available concerning MRSA/MSSA (methicillin-susceptible *S. aureus*) nasal carriage in healthy individuals in the broader community and/or in contact with animals, as well as in animals. The aim of this study was to investigate the prevalence and molecular epidemiology of MSSA/MRSA nasal carriage in different livestock animal species, as well as in humans in contact with them, in different regions of Algeria.

## 2. Results

### 2.1. Prevalence of Nasal Colonization with S. aureus

A total of 120 putative isolates of *S. aureus* were identified, of which 118 were confirmed by MALDI–TOF–MS. Nasal carriage was significantly more frequent in camels (*n* = 24; 53%) (*p* = 0.003) compared to horses (*n* = 5; 15.2%) and cattle (*n* = 6; 15%), followed by humans (*n* = 10; 50%), monkeys (*n* = 1; 50%) and sheep (*n* = 19; 44.2%). Among humans, cattle farmers (*n* = 5) were more colonized than horse owners/riders (*n* = 4), camel keepers (*n* = 1) and veterinarians (*n* = 1) (*p* = not significant). No regional effect on colonization frequency was observed in camels, but sheep from southern provinces were significantly more colonized compared to other regions (*p* = 0.03).

### 2.2. Antimicrobial Resistance

The antimicrobial resistance profiles of the tested *S. aureus* strains are summarized in Table 1.

All the isolates were susceptible to ceftobiprole, tobramycin, gentamicin, ofloxacin, fosfomycin, rifampicin, cotrimoxazole, quinopristin/dalfopristin, linezolid and glycopeptides. Resistance to penicillin G was significantly more prevalent in monkeys and horses than in humans, sheep, camels and cattle (*p* = 0.0001). Strains from monkeys, cattle and horses were fully sensitive to cefoxitin, while those from sheep were more resistant than those from camels (*p* = 0.0009). Nine *S. aureus* isolates (7.6%) were identified as MRSA. Three were isolated from camels (4.4% of the isolates) and six from sheep (9.3% of isolates). They showed co-resistance to kanamycin (for all the MRSA isolates) and fusidic acid (for one MRSA isolate). MRSA-positive camels belonged to the same herd, whilst MRSA-positive sheep were from three different flocks from Constantine. No human harbored MRSA strains.

### 2.3. CCs Distribution

**Figure 1:**
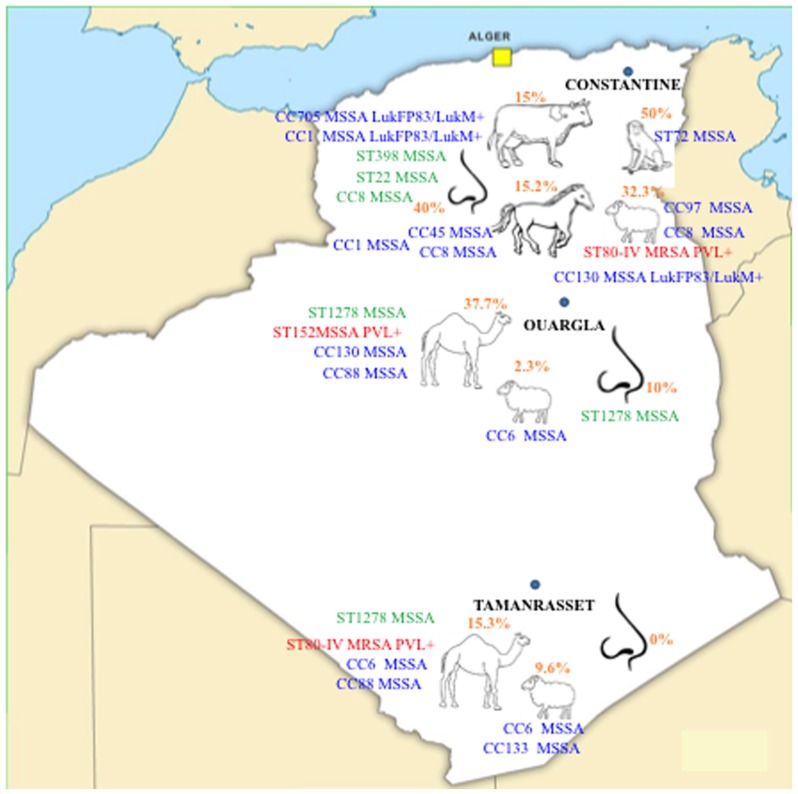
Main *S. aureus* clones circulating in Algeria among animals and humans in contact with them. Epidemic PVL+ clones are noted in red. Clones with potential animal origin are noted in green. The other clones are noted in blue.

DNA microarray analysis was carried out for a subset of 66 *S. aureus* isolates to determine their genetic diversity (12 from humans, 23 from camels, 19 from sheep, six from cattle, five from horses and one from a monkey). The main results of the CCs distribution are presented in Figure 1.

They were assigned to 15 CCs and 19 distinct strains (Table 2).

Camel *S. aureus* isolates clustered mainly within CC130, ST1278 (in association with a strain from a camel keeper) and CC88, the latter of which was exclusive to camels. Ovine strains grouped within CC6, ST80 and CC130 (together with those from camels), CC97 (with cattle) and CC8 (with strains from horses, a horse rider, horse workers and a cattle farmer), while ST133 was specific to sheep. Cattle isolates clustered specifically within CC705, CC15 (with isolates from a horse worker, a horse rider and a cattle farmer) and CC1 (with those from horses). ST152 and ST30 were specific to camels, ST398 and ST22 to humans (horse worker and cattle farmer, respectively). CC45 was represented by a single horse strain and ST72 by a unique strain from a monkey. The clustered intermixed isolates demonstrated an extremely close clonality and exhibited slight variations that were not influenced by their origins (species, farms or geographical regions).

### 2.4. Virulence Genes

All the virulence gene results of the studied strains are presented in Table 3.

*agr*III was significantly (*p* < 0.0001) predominant in all isolates (*n* = 31; 47.0%). Some of the microbial surface components recognizing adhesive matrix molecules (MSCRAMMs) genes were differently distributed among human and animal isolates, but the difference reached significance only for *fib* (*p* = 0.007). The majority of human isolates harbored the *cap5* gene (66.7%), while *cap8* was significantly more observed in animal isolates (72.2%) (*p* = 0.017). Interestingly, the PVL-encoding genes (*n* = 7) were only detected in camels and sheep. Other toxinogenic genes were frequently detected: *etD* was detected in eight strains (six from animals and two from humans), whilst the *edinB* gene (*n* = 18) was more commonly harbored by animal isolates (*n* = 16) than human ones (*n* = 2). This gene was found to be associated to *etD* in all MRSA and ST398 isolates. *tst1* also had a high prevalence (*n* = 14) with a similar repartition in animal (22.2%) and human (16.7%) isolates. 

### 2.5. Resistance Genes

The prevalence of resistance determinants is summarized in Table 3. 

Analysis of the resistance genes conformed to conventional susceptibility data. All MRSA isolates were detected by cefoxitin test and were positive for *mecA* and SCC*mec* cassettes by DNA arrays. No *mecC* gene was detected. The *aphA3* gene was found in all MRSA strains in addition to four MSSA strains (two animal and two human isolates). The *fosB* gene was significantly more prevalent in human isolates than in animal ones (*p* = 0.004). No *van* genes were detected, in agreement with the in vitro susceptibility data.

## 3. Discussion

To our knowledge, this study highlights for the first time the presence of PVL+ *S. aureus* isolates in livestock in Algeria. First, we detected isolates belonging to the ST80-MRSA clone in healthy sheep and camels. This clone has previously been found in sheep nasal swabs in Tunisia [8] and in retail camel meat in Saudi Arabia [9]. Positive sheep and camels were respectively from Constantine (in the northeast of Algeria) and Tamanrasset (in the south), located 2050 km apart (Figure 1). This suggests that this clone has already spread across the country and strengthens the hypothesis of its wide dissemination in North Africa. This clone occurs commonly in humans in Europe, North Africa, sub-Saharan Africa and the Middle East [7,10]. In Algeria it is the leading MRSA clone [11]. The presence of this clone even in very remote populations, such as the Gabonese Babongo Pygmies, underlies the dispersal of this MRSA-ST80 clone [12]. Moreover, our findings highlight the presence of the ST152 PVL+ clone. This clone is particularly frequent and widespread in West Africa (40–60% of the *S. aureus* strains) [7] but was never before described in Algeria. Interestingly, Mali borders part of southern Algeria and the ST152 PVL+ MSSA we have identified in our study was found in a camel from a southern province. This suggests that this clone is already established in camels and humans in this region. Malian refugees crossing borders with their animals are an example of human movements. It was previously described from nasal carriage in different animal species in sub-Saharan Africa [13]. Finally, our work also described for the first time the presence of MSSA-ST1278 in isolates from camels in Ouargla and Tamanrasset provinces, as well as a camel keeper in Ouargla province. This clone is a rarely and poorly characterized singleton of the CC1 lineage. It has been described in a very small number of human samples in Palestine, Israel and France [14,15,16].

A high variety of CCs have been detected in animals and humans. The discovery of classical human CCs (e.g., CC1, CC8, CC15, CC30, C45) in animals suggests a possible transmission from humans in our study region as previously evoked [17]. However, the detection of CCs specific to animals in humans (CC97, CC130, CC133, ST398 and ST705) indicates an acquisition through occupational contact [18]. The transmission seems to be more complex with the presence of several reservoirs (environment, other animal species or animals from the same species) [19]. For example, ST398 was found in a horse worker and a veterinarian in the Constantine region, which could suggest the possession of this livestock-associated lineage by further animals (e.g., horses and poultry) not screened in the study.

In animals, the prevalence of *S. aureus* nasal carriage in the literature varies between the species: 7.9% in horses, 46.5% in cattle, 53% in camels and 58% in monkeys [20,21,22]. In the present study, prevalence in camels (53%) was higher than previously reported in Jordan (13.7%) and Qatar (43.9%), and comparable to a study in Saudi Arabia (56.3%) [23,24,25]. In sheep, the prevalence varies from 29% in France to 44.8% in Tunisia [8,26]. Nasal carriage of MRSA was low in our animal panels: only sheep (9.3%) and camels (4.4%) harbored it. This carriage remains rare in other countries (2.8% in sheep, 2.0% in camels and 16.8% in cows in Nigeria; 3% in sheep in Tunisia; and 0.5% in horses in Belgium), except in Saudi Arabia where 33.3% of camels and 19.4% of cattle carried MRSA strains [8,21,25].

Some *S. aureus* genes have been demonstrated to have a human specificity (*sak*, *chp*, *scn*, *hlb*, *sea*) and their absence may be a valuable indicator of *S. aureus* livestock adaptation [27]. We confirmed this trend with absence of these markers in animal isolates. Interestingly, isolates of the ST80 cluster were tested positive to *sak* and *scn* genes, which confirm their human origin, but one ST80 isolate from a sheep was negative to all these genes, suggesting an adaptation to its animal host. Other studies have revealed that some STs (ST97 and ST151 of CC705), isolated from cattle and humans, have evolved from the same ancestors through acquisition of foreign DNA that are absent from *S. aureus* of human origin [28]. This ability to gain DNA carried on mobile genetic elements renders animal-specific lineages fit to colonize humans, and also makes them more virulent and resistant. For instance, horizontal transfer of a chromosomal cassette confers the ability to produce toxins such as PVL. This toxin is a crucial cytotoxic virulence factor in serious infections (necrotizing pneumonia, skin and soft tissue infections) and its occurrence among MSSA strains is endemic to Africa [7,14]; However, PVL-positive strains are rare in animals [5]. We identified *lukF/S*-PV genes in six ST80-MRSA strains from six sheep and two camels, in addition to a ST152-MSSA isolate from a camel. Van Duijkeren et al. reported the presence of *lukF/S*-PV genes in ewe isolates, but not in goats [29]. It was also described in Africa in cows, goats, donkeys, non-human primates and pigs [8,18,30]. The high pathogenicity of these isolates in humans requires follow-up studies in animals, where their presence must be taken into consideration by public health professionals, as they can serve as reservoirs for this virulence factor. Similarly, the animal isolates of our study harbored other toxinogenic genes (*tst1*, *edinB*, *etD*), illustrating the dissemination of strains harboring these highly pathogenic strains relevant for human health. Interestingly, *tst1* was associated with *sec* and *sel* in 10 animals (sheep, camels and cattle) isolates and one human isolate. Moreover, this combination of toxinogenic genes was associated with *lukM* and *lukDE* genes exclusively in ruminant isolates. These genes may either be located in the same or associated genetic elements, or may be otherwise related to the identified clonal lineages. The *etD* gene was identified in all animals harboring ST80-MRSA isolates. Its association with *edinB* is well known. The phage-borne *lukF-PV(P83)/lukM* locus, encoding a bi-component leukotoxin highly active against bovine neutrophils, was identified in isolates only from ruminants. This locus demonstrates host specificity as it has been frequently detected in isolates from livestock-associated lineages CC133 and CC479, but sporadically in CC30 and CC97 [19]. The high prevalence of *lukM* among sheep isolates (11%) in comparison to camels and cattle isolates (4.5% for each) may be explained by *S. aureus* adaptation to the higher resistance of small ruminants’ polymorphonuclear cells and macrophages to this leukotoxin [31].

Finally, very few enterotoxins were present in the screened isolates. The *sea* gene is carried by prophage *Sa3mu* alone, or by *Sa3mw* in combination with other genes (*seg* and *sek*) [32]. We detected it alone in a human strain, which implicitly means its carriage on the prophage *Sa3mu*. In contrast with previous studies which demonstrated that *sem*, *sen*, *seg*, *sei* and *seo* predominated in *S. aureus* from animal hosts [33], we identified them only in seven (12.9%) of our isolates. Their absence could be related to the loss of the mobile elements they are carried on. However, the occurrence of multiple toxinogenic genes in *S. aureus* is considered rare, which can explain the absence of many of these genes. These strains may pose a public-health risk to consumers, as they can contaminate food of animal origin and cause food poisoning.

## 4. Conclusions

This study showed the dispersal of some highly pathogenic clones ST152-MSSA-PVL+ and ST-80-MRSA-PVL+, as well as the identification of isolates with toxinogenic virulence factors (*lukPV*, *tst1*, *edinB*) in animals. It also suggested the ability of some clones to cross the species barrier and jump between humans and several animal species. Animal movements through the desert to the coastal provinces increase the dissemination risk of these clones. Our findings support the hypothesis that animals are potent reservoirs of multidrug-resistant and toxinogenic bacteria.

## 5. Materials and Methods

### 5.1. Study Population

From September 2015 to February 2016, different animal species and humans in contact with them were screened for *S. aureus* nasal carriage. Forty-five Dromedary camels (belonging to 7 herds from 2 southern Algerian provinces: Tamanrasset and Ouargla), 43 sheep (at 4 farms and the slaughterhouse of Constantine, a flock from Tamanrasset and another from Ouargla), 40 cattle (at 3 farms and the slaughterhouse of Constantine), 33 horses (at the equestrian club and a private horse stable at Constantine) and two monkeys (belonging to the private horse stable) were enrolled, as well as 20 consenting healthy persons in contact with these animals: cattle farmers, camel keepers, horse owners, horse riders, horse workers and veterinarians.

### 5.2. Specimen Collection and Bacterial Isolation

For each individual (animal or human), both nares/nostrils were sampled. A sterile cotton-tipped swab was inserted about 5 to 10 cm into the nasal cavities (sheep, cattle, horses and camels) or into anterior nares (humans and monkeys), and gently rubbed against the mucosa for 5 to 10 s. Swabs were directly inoculated into brain–heart infusion broth, and then cultured on Baird Parker-RPF agar (Conda Pronadisa, Madrid, Spain). Five fresh *S. aureus*-like colonies from each individual were selected for presumptive conventional phenotypic and biochemical tests.

### 5.3. Identification and Susceptibility Testing

The identification was confirmed by a MALDI–TOF system (bioMérieux, Marcy l’Etoile, France). Susceptibility to antimicrobial agents was determined by the disk diffusion method on Mueller–Hinton agar according to recommendations of EUCAST-CASFM [34]. MRSA isolates were identified using cefoxitin disks (30 μg).

### 5.4. Oligonucleotide DNA Arrays and Genotyping

Each *S. aureus* isolate collected during the study was analyzed at the INSERM laboratory in Nîmes, France. Isolates belonging to the same individual and showing similar drug resistance patterns were assumed to be derived from the same strain; thus, a subset of 66 non-repetitive isolates was selected for molecular analyses. The Alere StaphyType DNA microarray was used according to protocols and procedures previously detailed [35]. The test was able to screen numerous markers simultaneously in 5 h. The DNA microarray covers 334 target sequences including the main virulence and resistance genes. Primer and probe sequences have been published previously [35]. DNA was extracted from each *S. aureus* strain and after amplification and hybridization, markers were identified. A digital picture of the microarray was taken and analyzed, using a dedicated reader and software (ALERE Technologies GmbH, Jena, Germany). The affiliation of isolates to clonal complexes (CCs) or sequence types (STs) as defined by MLST [35] was determined by an automated comparison of hybridization profiles against a collection of reference strains previously characterized [35].

### 5.5. Statistical Analysis

Differences in nasal carriage, resistance or virulence genes profiles were assessed by a chi-square test or Fisher’s exact two-tailed test (when n < 5) using the statistical software GraphPad Instat prism ver.6.04 (GraphPad Software Inc., San Diego, CA, USA. 2014). Statistical significance was set at a *p* ≤ 0.05 and 95% CI.

## Figures and Tables

**Table 1:** Resistance profiles of *Staphylococcus aureus* strains isolated from nasal samples of livestock and humans in contact with them in three Algerian provinces.

Antibiotics	Origin of Isolates						
	Humans (*n* = 27) n (%)	Horses (*n* = 11) n (%)	Camels (*n* = 41) n (%)	Cattle (*n* = 8) n (%)	Sheep (*n* = 30) n (%)	Monkeys (*n* = 1) n (%)	Total Animals (*N* = 91) n (%)
Penicillin G	24 (88.8)	11 (100)	14 (34.1)	2 (25.0)	16 (53.3)	1 (100)	44 (48.4)
Cefoxitin	0 (0)	0 (0)	3 (7.3)	0 (0)	6 (20.0)	0 (0)	9 (9.9)
Erythromycin	6 (22.2)	0 (0)	0 (0)	0 (0)	2 (6.7)	0 (0)	2 (2.2)
Ceftobiprole	0 (0)	0 (0)	0 (0)	0 (0)	0 (0)	0 (0)	0 (0)
Clindamycin	4 (14.8)	0 (0)	0 (0)	0 (0)	2 (6.7)	0 (0)	2 (2.2)
Quinupristin/Dalfopristin	0 (0)	0 (0)	0 (0)	0 (0)	0 (0)	0 (0)	0 (0)
Kanamycin	3 (11.1)	6 (54.5)	3 (7.3)	0 (0)	3 (10.0)	0 (0)	12 (13.2)
Tobramycin	0 (0)	0 (0)	0 (0)	0 (0)	0 (0)	0 (0)	0 (0)
Gentamicin	0 (0)	0 (0)	0 (0)	0 (0)	0 (0)	0 (0)	0 (0)
Minocycline	3 (11.1)	6 (54.5)	0 (0)	0 (0)	0 (0)	0 (0)	6 (6.6)
Ofloxacin	0 (0)	0 (0)	0 (0)	0 (0)	0 (0)	0 (0)	0 (0)
Fucidic acid	0 (0)	0 (0)	2 (4.9)	0 (0)	0 (0)	0 (0)	2 (2.2)
Linezolid	0 (0)	0 (0)	0 (0)	0 (0)	0 (0)	0 (0)	0 (0)
Fosfomycin	0 (0)	0 (0)	0 (0)	0 (0)	0 (0)	0 (0)	0 (0)
Rifampicin	0 (0)	0 (0)	0 (0)	0 (0)	0 (0)	0 (0)	0 (0)
Cotrimoxazole	0 (0)	0 (0)	0 (0)	0 (0)	0 (0)	0 (0)	0 (0)

**Table 2:** Clonal complex distribution of the *Staphylococcus aureus* strains isolated from nasal samples of livestock (A) and humans (H) in contact with them in three Algerian provinces.

CC	Clone Assignment	Leukocidin Genes Content	Origin of Isolates							*p*
			Human (*n* = 12) n (%)	Horses (*n* = 5) n (%)	Camels (*n* = 23) n (%)	Cattle (*n* = 6) n (%)	Sheep (*n* = 19) n (%)	Monkeys (*n* = 1) n (%)	Total Animals (*N* = 54) n (%)	H vs A
CC130	ST130	-	0 (0)	0 (0)	6 (26.1)	0 (0)	0 (0)	0 (0)	6 (11.1)	NS
	ST130-MSSA	LukF-P83/LukM+	0 (0)	0 (0)	0 (0)	0 (0)	3 (15.8)	0 (0)	3 (5.6)	NS
CC1	ST1-MSSA	-	0 (0)	2 (40.0)	0 (0)	0 (0)	0 (0)	0 (0)	2 (3.7)	NS
	ST1-MSSA	LukF-P83/LukM+	0 (0)	0 (0)	0 (0)	1 (16.7)	0 (0)	0 (0)	1 (1.9)	NS
	ST1278-MSSA	-	1 (8.3)	0 (0)	4 (17.4)	0 (0)	0 (0)	0 (0)	4 (7.4)	NS
CC8	ST8-MSSA	-	4 (33.3)	2 (40.0)	0 (0)	0 (0)	4 (21.1)	0 (0)	6 (11.1)	NS
	ST72-MSSA	-	0 (0)	0 (0)	0 (0)	0 (0)	0 (0)	1	1 (1.9)	NS
CC6	ST6-MSSA	-	0 (0)	0 (0)	3 (13.0)	0 (0)	4 (21.1)	0 (0)	7 (13.0)	NS
CC80	ST80-MRSA IV	LukS-PV/LukF-PV+	0 (0)	0 (0)	2 (8.7)	0 (0)	4 (21.1)	0 (0)	6 (11.1)	NS
CC88	ST88-MSSA	-	0 (0)	0 (0)	6 (26.1)	0 (0)	0 (0)	0 (0)	6 (11.1)	NS
CC97	ST97-MSSA	-	0 (0)	0 (0)	0 (0)	1 (16.7)	2 (10.5)	0 (0)	3 (5.6)	NS
CC15	ST15-MSSA	-	3 (25.0)	0 (0)	0 (0)	2 (33.3)	0 (0)	0 (0)	2 (3.7)	0.038
CC133	ST133-MSSA	-	0 (0)	0 (0)	0 (0)	0 (0)	2 (10.5)	0 (0)	2 (3.7)	NS
CC705	ST705	LukF-P83/LukM+	0 (0)	0 (0)	0 (0)	2 (33.3)	0 (0)	0 (0)	2 (3.7)	NS
CC152	ST152-MSSA	LukS-PV/LukF-PV+	0 (0)	0 (0)	1 (4.3)	0 (0)	0 (0)	0 (0)	1 (1.9)	NS
CC30	ST30-MSSA	-	1 (8.3)	0 (0)	1 (4.3)	0 (0)	0 (0)	0 (0)	1 (1.9)	NS
CC45	ST45-MSSA	-	0 (0)	1 (20.0)	0 (0)	0 (0)	0 (0)	0 (0)	1 (1.9)	NS
CC398	ST398-MSSA	-	2 (16.7)	0 (0)	0 (0)	0 (0)	0 (0)	0 (0)	0 (0)	0.030
CC22	ST22-MSSA	-	1 (8.3)	0 (0)	0 (0)	0 (0)	0 (0)	0 (0)	0 (0)	NS

**Table 3:** Main virulence and resistance gene profiles in *S. aureus* isolated from nasal samples of livestock (A) and humans (H) in contact with them in three Algerian provinces.

Virulence Genes	Origin of Isolates							*p*
	Human (*n* = 12) n (%)	Horses (*n* = 5) n (%)	Camels (*n* = 23) n (%)	Cattle (*n* = 6) n (%)	Sheep (*n* = 19) n (%)	Monkeys (*n* = 1) n (%)	Total Animals (*N* = 54) n (%)	H vs. A
Virulence genes								
Enterotoxins								
*sea*	0 (0)	0 (0)	0 (0)	1 (16.7)	0 (0)	0 (0)	1 (1.9)	NS
*seb*	0 (0)	0 (0)	0 (0)	0 (0)	0 (0)	0 (0)	0 (0)	NS
*egc cluster* *	2 (16.6)	1 (20)	1 (4.3)	2 (33.3)	0 (0)	1 (100)	5 (9.2)	NS
*seg*	2 (16.6)	1 (20)	1 (4.3)	2 (33.3)	0 (0)	1 (100)	5 (9.2)	NS
*seh*	1 (8.3)	2 (40)	4 (17.4)	1 (16.7)	0 (0)	0 (0)	7 (12.9)	NS
*sek*	0 (0)	0 (0)	0 (0)	0 (0)	0 (0)	0 (0)	0 (0)	NS
*seq*	0 (0)	0 (0)	0 (0)	0 (0)	0 (0)	0 (0)	0 (0)	NS
Other toxins								
*tst*	2 (16.6)	0 (0)	3 (13)	2 (33.3)	7 (36.8)	0 (0)	12 (22.2)	NS
*etA*	0 (0)	0 (0)	0 (0)	0 (0)	0 (0)	0 (0)	0 (0)	NS
*etB*	0 (0)	0 (0)	0 (0)	0 (0)	0 (0)	0 (0)	0 (0)	NS
*etD*	2 (16.6)	0 (0)	2 (8.7)	0 (0)	4 (21.0)	0 (0)	6 (11.1)	NS
*edinB*	2 (16.6)	0 (0)	9 (39.1)	0 (0)	7 (36.8)	0 (0)	16 (29.6)	NS
*lukS-PV/lukF*-PV	0 (0)	0 (0)	3 (13)	0 (0)	4 (21)	0 (0)	7 (13)	NS
*lukDE*	12 (100)	4 (80)	10 (43.4)	5 (83.3)	10 (52.6)	1 (100)	30 (55.5)	0.002
Hemolysins								
*hla*	11 (91.6)	3 (60)	21 (91.3)	6 (100)	19 (100)	1 (100)	50 (92.5)	NS
*hld*	12 (100)	5 (100)	23 (100)	6 (100)	19 (100)	1 (100)	54 (100)	NS
*hlgA*	12 (100)	5 (100)	23 (100)	6 (100)	19 (100)	1 (100)	54 (100)	NS
*hlg*	5 (41.6)	1 (20)	7 (30.4)	2 (33.3)	4 (21)	1 (100)	15 (27.7)	NS
*hlgv*	10 (83.3)	4 (80)	20 (86.9)	6 (100)	19 (100)	1 (100)	50 (92.5)	NS
MSCRAMMs								
*bbp*	12 (100)	5 (100)	21 (91.3)	4 (66.6)	17 (89.4)	1 (100)	48 (88.8)	NS
*cna*	3 (25)	3 (60)	8 (34.7)	1 (16.7)	4 (21)	0 (0)	16 (29.6)	NS
*ebpS*	12 (100)	5 (100)	23 (100)	6 (100)	19 (100)	1 (100)	54 (100)	NS
*clfA*	12 (100)	5 (100)	23 (100)	6 (100)	19 (100)	1 (100)	54 (100)	NS
*clfB*	12 (100)	5 (100)	23 (100)	6 (100)	19 (100)	1 (100)	54 (100)	NS
*fib*	7 (58.3)	3 (60)	21 (91.3)	6 (100)	19 (100)	1 (100)	50 (92.5)	0.007
*fnbA*	12 (100)	5 (100)	23 (100)	6 (100)	19 (100)	1 (100)	54 (100)	NS
*fnbB*	10 (83.3)	4 (80)	20 (86.9)	4 (66.6)	12 (63.1)	1 (100)	41 (75.9)	NS
Capsule components								
*cap*5	8 (66.6)	2 (40)	5 (21.7)	1 (16.7)	6 (31.5)	1 (100)	15 (27.7)	0.017
*cap*8	4 (33.3)	3 (60)	18 (78.2)	5 (83.3)	13 (68.4)	0 (0)	39 (72.2)	0.017
*icaA*	12 (100)	5 (100)	23 (100)	6 (100)	19 (100)	1 (100)	54 (100)	NS
*icaC*	12 (100)	5 (100)	23 (100)	6 (100)	19 (100)	1 (100)	54 (100)	NS
*icaD*	12 (100)	5 (100)	23 (100)	6 (100)	19 (100)	1 (100)	54 (100)	NS
Other virulence factors								
*chp*	7 (58.3)	1 (20)	0 (0)	2 (33.3)	0 (0)	0 (0)	3 (5.5)	0.0001
*scn*	8 (66.6)	1 (20)	7 (30.4)	2 (33.3)	3 (15.8)	0 (0)	13 (24)	0.012
Accessory gene regulators								
*agr1*	7 (58.33)	3 (60)	4 (17.4)	1 (16.7)	12 (63.1)	1 (100)	21 (38.8)	NS
*agr2*	3 (25)	0 (0)	0 (0)	4 (66.6)	0 (0)	0 (0)	4 (7.4)	NS
*agr3*	2 (16.6)	2 (40)	19 (82.6)	1 (16.7)	7 (36.8)	0 (0)	29 (53.7)	0.026
*agr4*	0 (0)	0 (0)	0 (0)	0 (0)	0 (0)	0 (0)	0 (0)	NS
Resistance genes								
*mecA*	0 (0)	0 (0)	2 (8.7)	0 (0)	4 (21.0)	0 (0)	6 (11.1)	NS
*mecC*	0 (0)	0 (0)	0 (0)	0 (0)	0 (0)	0 (0)	0 (0)	NS
*blaZ*	11 (91.6)	5 (100)	5 (21.7)	2 (33.3)	5 (26.3)	1 (100)	18 (33.3)	0.001
*ermA*	1 (8.3)	0 (0)	0 (0)	0 (0)	0 (0)	0 (0)	0 (0)	NS
*ermC*	1 (8.3)	0 (0)	0 (0)	0 (0)	0 (0)	0 (0)	0 (0)	NS
*aacA-aphD*	0 (0)	0 (0)	0 (0)	0 (0)	0 (0)	0 (0)	0 (0)	NS
*tetM*	2 (16.6)	2 (40)	0 (0)	0 (0)	0 (0)	0 (0)	2 (3.7)	NS
*tetK*	3 (25)	4 (80)	0 (0)	2 (33.3)	5 (26.3)	0 (0)	11 (20.3)	NS
*fosB*	8 (66.6)	2 (40)	1 (4.3)	2 (33.3)	6 (31.5)	1 (100)	12 (22.2)	0.004

* *egc* cluster corresponds to *seg*, *sei*, *sem*, *sen* and *seo* genes.
